# Peripheral microcirculatory alterations are associated with the severity of acute respiratory distress syndrome in COVID-19 patients admitted to intermediate respiratory and intensive care units

**DOI:** 10.1186/s13054-021-03803-2

**Published:** 2021-11-08

**Authors:** Jaume Mesquida, A. Caballer, L. Cortese, C. Vila, U. Karadeniz, M. Pagliazzi, M. Zanoletti, A. Pérez Pacheco, P. Castro, M. García-de-Acilu, R. C. Mesquita, D. R. Busch, T. Durduran, the HEMOCOVID-19 Consortium, Turgut Durduran, Marco Pagliazzi, Lorenzo Cortese, Marta Zanoletti, Umut Karadeniz, Jaume Mesquida, Alba Caballer, Sara Nogales, Cristina Espinal, Guillem Gruartmoner, Puri Pérez Terán, Clara Vilà, Lucía Picazo, Ricard Ferrer, Marina García De Acilu, Luis Chiscano, Abraham Mera, Pedro Castro, Adrián Téllez, Sara Fernández, Ana Matas, Fernando Fuentes, Isabel Serra, David Romero, Francesc Font, Tim Myers, David R. Busch, Siddharth Dave, Sreekanth Cheruku, Christopher Choi, Peiman Lahsaei, DaiWai Olson, Argelia Pérez Pacheco, Rosa María Quispe Siccha, Eduardo Liceaga, Félix Jerandy Monte De Oca Hernández, Bruno Adler Maccagnan Pinheiro Besen, Leandro Utino Taniguchi, Pedro Vitale Mendes, Rickson Coelho Mesquita, Andrés Fabián Quiroga Soto, Italo Karmann Aventurato, Laís Bacchin de Oliveira, Lilian Elisabete Bernardes Delazari, Lígia dos Santos, Roceto Ratti, Antonio Luis Eiras Falcão, Judith Marin-Corral, Raúl Serrano-Loyola, Verónica Carbajal-Robles, Enrique Santillan-Aguayo, Melvin Parada-Guzmán, Rodrigo Menezes-Forti, Luis Bacchin, Gabriela Lívio-Emidio

**Affiliations:** 1 Àrea de Crítics, Parc Taulí Hospital Universitari, Parc Taulí, 1, 08208 Sabadell, Spain; 2 ICFO-Institut de Ciències Fotòniques, The Barcelona Institute of Science and Technology, Castelldefels, Barcelona, Spain; 3 Servei de Medicina Intensiva, Parc Salut Mar Hospital, Barcelona, Spain; 4 Hospital General de México, Mexico, Mexico; 5 Medical Intensive Care Unit, Hospital Clínic de Barcelona, IDIBAPS, Barcelona, Spain; 6 Intensive Care Department, Hospital Universitari Vall d’Hebron, Barcelona, Spain; 7 Institute of Physics, University of Campinas, Campinas, Brazil; 8 University of Texas Southwestern Medical Center, Dallas, TX USA; 9 Institució Catalana de Recerca i Estudis Avançats (ICREA), Barcelona, Spain

**Keywords:** COVID-19, Microcirculation, Near-infrared spectroscopy, Endothelial dysfunction

## Abstract

**Background:**

COVID-19 is primarily a respiratory disease; however, there is also evidence that it causes endothelial damage in the microvasculature of several organs. The aim of the present study is to characterize in vivo the microvascular reactivity in peripheral skeletal muscle of severe COVID-19 patients.

**Methods:**

This is a prospective observational study carried out in Spain, Mexico and Brazil. Healthy subjects and severe COVID-19 patients admitted to the intermediate respiratory (IRCU) and intensive care units (ICU) due to hypoxemia were studied. Local tissue/blood oxygen saturation (StO_2_) and local hemoglobin concentration (THC) were non-invasively measured on the forearm by near-infrared spectroscopy (NIRS). A vascular occlusion test (VOT), a three-minute induced ischemia, was performed in order to obtain dynamic StO_2_ parameters: deoxygenation rate (DeO_2_), reoxygenation rate (ReO_2_), and hyperemic response (H_AUC_). In COVID-19 patients, the severity of ARDS was evaluated by the ratio between peripheral arterial oxygen saturation (SpO_2_) and the fraction of inspired oxygen (FiO_2_) (SF ratio).

**Results:**

Healthy controls (32) and COVID-19 patients (73) were studied. Baseline StO_2_ and THC did not differ between the two groups. Dynamic VOT-derived parameters were significantly impaired in COVID-19 patients showing lower metabolic rate (DeO_2_) and diminished endothelial reactivity. At enrollment, most COVID-19 patients were receiving invasive mechanical ventilation (MV) (53%) or high-flow nasal cannula support (32%). Patients on MV were also receiving sedative agents (100%) and vasopressors (29%). Baseline StO_2_ and DeO_2_ negatively correlated with SF ratio, while ReO_2_ showed a positive correlation with SF ratio. There were significant differences in baseline StO_2_ and ReO_2_ among the different ARDS groups according to SF ratio, but not among different respiratory support therapies.

**Conclusion:**

Patients with severe COVID-19 show systemic microcirculatory alterations suggestive of endothelial dysfunction, and these alterations are associated with the severity of ARDS. Further evaluation is needed to determine whether these observations have prognostic implications. These results represent interim findings of the ongoing HEMOCOVID-19 trial.

*Trial registration* ClinicalTrials.gov NCT04689477. Retrospectively registered 30 December 2020.

**Supplementary Information:**

The online version contains supplementary material available at 10.1186/s13054-021-03803-2.

## Background

Since its first identification in December 2019, over 190 million people have been diagnosed of severe acute respiratory syndrome coronavirus 2 (SARS-CoV-2) infection worldwide, and Coronavirus disease 2019 (COVID-19) has been responsible for over 4 million deaths [1]. Although 80% of those infected with COVID-19 will develop only mild symptoms, critically ill patients, presenting with acute respiratory hypoxemic failure, account for up to 15% of cases [2, 3]. Overall mortality rates range from 1 to 4%, but in severe cases requiring intensive care, mortality increases to 30–50% [4, 5].

Although COVID-19 typically begins as an infection of the upper airway, it can progress to severe respiratory disease, including acute respiratory distress syndrome (ARDS). However, SARS-CoV-2 has been detected in multiple organs and some authors suggest COVID-19 should be considered a systemic vascular disease, mainly affecting the vascular endothelium [6, 7]. The main suspected mechanisms for endothelial dysfunction (ED) are the direct cytopathic effect of the virus and the effect of inflammation mediators resulting from the host immune response. Postmortem examinations have shown three main features at the vascular level: (I) endothelial damage, with both viral inclusions and endothelial inflammation including monocellular cell infiltrate and lymphocytic endotheliitis; (II) extensive vascular thrombosis; and (III) abnormal vascular architecture [6, 7]. Consequently, monitoring endothelial function emerges as a potential biomarker of COVID-19 severity for prognostic purposes or monitoring the effect of new treatment options [8, 9].

To date, optical technologies capable of monitoring the endothelial status have been used in clinical research with a great deal of promise. In particular, near-infrared spectroscopy (NIRS) provides a non-invasive, portable, assessment of tissue oxygenation. The evaluation of microcirculatory health by NIRS technologies has repeatedly demonstrated its prognostic value in other conditions where ED plays a major role, such as sepsis [10–12]. Therefore, we designed a preliminary study aiming at characterizing the microvascular reactivity in peripheral skeletal muscle in patients with COVID-19-associated ARDS entering critical care areas.

## Material and methods

### Design and setting

A prospective, multicenter, observational study carried out in Spain, Mexico, and Brazil. The results herein report data from six hospitals out of eight that are currently participating in the HEMOCOVID-19 Consortium. The study was approved by the Ethics Committee of each participating center and registered to Clinical Trials (ClinicalTrials.gov NCT04689477). This study is presented following the STROBE recommendations for reporting observational studies [13].

### Subjects

The study included two different groups: (1) Healthy volunteers; and (2) Severe COVID-19 patients:

1. Healthy volunteers: Healthy adult subjects, with no previous history of disease or recent medications that could affect blood circulation.
2. Adult patients with COVID-19-associated ARDS admitted to the Intermediate Respiratory Care Unit (IRCU) or the Intensive Care Unit (ICU), within the first week of admission.

Exclusion criteria included evidence of venous thrombosis in the upper limbs, and hematoma or skin lesions in the forearm that could hinder placement of NIRS sensor probe. Hemodynamic unstable patients were not included, defined as uncorrected arterial hypotension and/or the need for active resuscitation interventions for optimizing blood pressure and/or cardiac output. The participation of subjects was voluntary, and informed consent was obtained from the patient or from the patients' legal representative.

### Variables

Patient demographic information, comorbidities, respiratory and hemodynamic status, and tissue oxygenation of peripheral skeletal muscle were collected. Respiratory, hemodynamic, and microcirculatory parameters were studied simultaneously.

- *Respiratory parameters*: Respiratory rate (RR), oxygenation status, and ventilator settings (if applicable). Arterial oxygen saturation via pulse-oximetry (SpO_2_) and the fraction of inspired oxygen administered (FiO_2_) were used to obtain the SF ratio (SpO_2_/FiO_2_). When arterial blood gas analysis was available, arterial partial pressure of oxygen (PaO_2_) and FiO_2_ were used to obtain the PF ratio (PaO_2_/FiO_2_).
- *Hemodynamic parameters*: Heart rate (HR) and blood pressure.
- *Tissue oxygenation*: Tissue oxygen saturation was measured continuously, and non-invasively, on peripheral skeletal muscle by means of a NIRS system (PortaMon, Artinis Medical Systems. Elst, The Netherlands). The PortaMon is a portable (8 × 4 × 1.5 cm) battery-operated NIRS device, which is remotely controlled via a Bluetooth connection. It utilizes three pairs of Light Emitting Diodes (LED, 760 nm and 850 nm) at three different distances from a photodetector (30, 35 and 40 mm). The spatial and wavelength dependence of optical attenuation enables calculation of local tissue blood oxygen saturation (StO_2_) and total hemoglobin concentration (THC) in a sample volume extending 1–2 cm below the skin at a sampling rate of 10 Hz.

### Study protocol

Once consent to participate was obtained, the operator recorded vital signs and clinically relevant information. The NIRS probe was placed on intact skin on the brachioradialis muscle of the forearm and never adjacent to a radial cannulation site. After securing the probe to provide a stable StO_2_ signal, a three-minute baseline period was recorded, followed by a Vascular Occlusion Test (VOT).

The VOT was performed as previously described [10, 14, 15]. Briefly, a blood pressure cuff was placed around the arm, proximal to the studied forearm. The cuff was rapidly inflated to 50 mmHg above the systolic pressure and kept inflated for 3-min, at which time, the cuff was rapidly deflated. The resulting deoxygenation (DeO_2_) and reoxygenation (ReO_2_) slopes were reported as change in O_2_ saturation over time. The hyperemic response following the reoxygenation was reported as an area under the curve (H_AUC_). The calculation of the NIRS-derived variables is described in Additional file 1. DeO_2_ provides information on the tissue metabolic rate, while both, ReO_2_ and H_AUC_ provide information on the microvascular reactivity of the measured tissue [10].

### Sample size calculation

Accepting an alpha risk of 0.05 and a beta risk of 0.2 in a two-sided test, and a control subject to patient ratio of 1:2, thirty-two subjects were necessary in the healthy volunteers group and seventy-two in the COVID-19 group to recognize as statistically significant a difference in ReO_2_ greater than or equal to 30%/min. According to published literature from our group and others the common standard deviation for ReO_2_ was assumed to be 50%/min [10–12, 14]. Sample size was calculated by means of the GRANMO Sample size and power calculator tool of the Institut Municipal d’Investigació Mèdica de Barcelona (IMIM) (https://www.imim.cat/ofertadeserveis/software-public/granmo/). We note that these are intermediate results of a larger study where we seek to detect even smaller variations and their association to different physiological/clinical variables.

### Statistical analysis

Statistical analysis was performed with SPSS v.20 (IBM Corporation). The normality of the distribution of the studied variables was tested using the Kolmogorov–Smirnov test. Continuous variables were expressed as mean ± standard deviation (SD), and categorical variables were expressed as absolute numbers and proportions (%). A descriptive analysis was performed. Comparisons between groups were performed with Chi-Square test for categorical variables and Student's *t* test for continuous variables with a normal distribution. Correlations were analyzed by means of the Pearson’s correlation test. ICU patients were classified into mild, moderate, and severe ARDS groups by applying SF ratio cutoffs as described by Rice et al. [16] (SF ratio cutoffs of 315, 235, and 144, respectively). Differences among ARDS severity groups and among different ventilatory support therapies were analyzed using the single-factor analysis of variance (ANOVA) test. A post hoc Tukey comparison test followed significant results for ANOVA. Linear regression models were used for testing associations between the studied variables and the severity of the respiratory disease. A two-tailed *p* value < 0.05 was considered to indicate statistical significance.

## Results

### Healthy volunteers versus COVID-19 patients

**Fig. 1:**
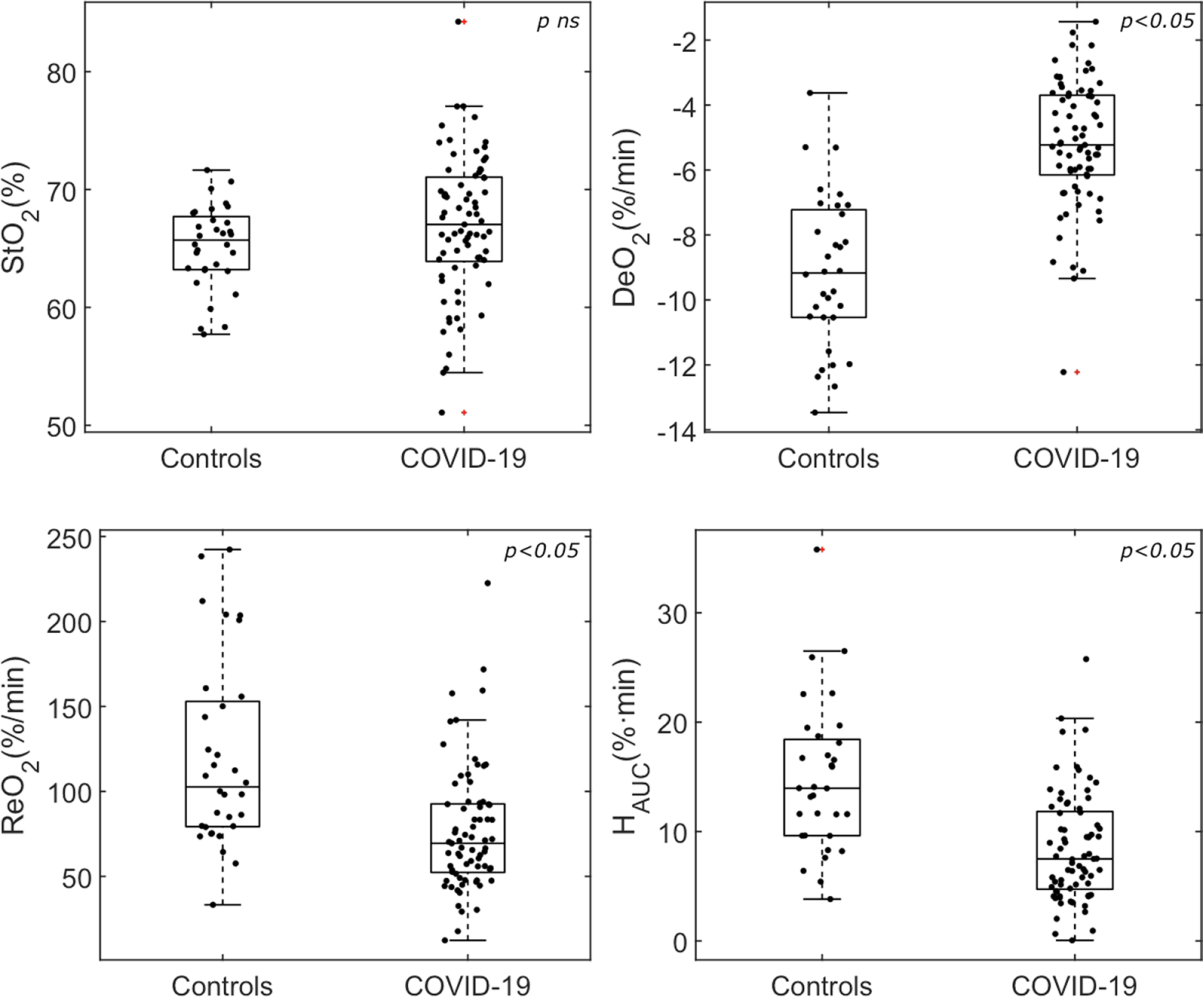
Box-plot and individual data points showing microcirculatory parameters in healthy volunteers and COVID-19 patients. Although baseline StO_2_ values did not differ, all dynamic StO_2_ indices were altered in COVID-19 patients, as compared to healthy controls

Thirty-two healthy controls and seventy-three COVID-19 patients were included between July 2020 and January 2021. Measurements done for training purposes at different centers were excluded. All consecutively studied COVID-19 patients with complete data sets were included in this preliminary analysis. The main demographic and clinical characteristics of the study sample are summarized in Table 1. COVID-19 patients were older (59 ± 13 vs. 34 ± 10, *p* < 0.001) and with a higher body mass index (BMI) (30 ± 5 vs. 23 ± 3, *p* < 0.001) than control subjects. Hemodynamic status was similar in both groups, except for the use of vasopressors in 13% of patients. Respiratory state differed in terms of RR and FiO_2_ (both higher in the patient group), while SpO_2_ was similar between groups. Baseline StO_2_ and THC did not differ between the two groups. Dynamic VOT-derived parameters were significantly impaired in the COVID-19 patient group, showing lower metabolic rate (DeO_2_), and diminished microvascular reactivity (ReO_2_ and H_AUC_) as compared to the healthy population (Fig. 1).

**Table 1:** Main characteristics of the studied healthy volunteers and critically ill COVID-19 patients at inclusion

	Healthy volunteers (*n* = 32)	COVID-19 patients (*n* = 73)
Age (years)	34 ± 10	59 ± 13*
Gender (male), *n* (%)	16 (50)	51 (70)*
BMI	23 ± 3	30 ± 5*
Days from hospitalization (*n*)	–	5 ± 4
Days from IRCU/ICU admission (*n*)	–	3 ± 3
APACHE II score	–	14 ± 8
*Pre-existing comorbidities* (*n*, %)		
Hypertension	–	33 (45)
Diabetes mellitus	–	17 (23)
Smoker	–	18 (25)
COPD	–	7 (10)
Temperature (°C)	36.3 ± 0.3	36.0 ± 0.7
HR (beats/min)	75 ± 13	77 ± 16
MAP (mmHg)	83 ± 9	86 ± 12
RR (resp/min)	18 ± 3	22 ± 5*
SpO_2_ (%)	97 ± 2	95 ± 4
FiO_2_ (%)	21 ± 0	54 ± 21*
StO_2_ (%)	65 ± 3	67 ± 6
THC (μM/L)	45 ± 11	43 ± 16
DeO_2_ (%/min)	− 9.1 ± 2.4	− 5.2 ± 2.0*
ReO_2_ (%/min)	120 ± 55	77 ± 37*
Hyperemia AUC (U)	14.8 ± 6.8	8.5 ± 5.0*

BMI, body mass index; IRCU, Intermediate Respiratory Care Unit; ICU, Intensive Care Unit; COPD, chronic obstructive pulmonary disease; HR, heart rate; MAP, mean arterial pressure; RR, respiratory rate; SpO_2_, pulse-oximetric oxygen saturation; FiO_2_, fraction of inspired oxygen; StO_2_, tissue oxygen saturation; THC, tissue hemoglobin concentration; DeO_2_, deoxygenation rate; ReO_2_, reoxygenation rate; AUC, area under the curve

^*^p < 0.05

### COVID-19 patient analysis

**Fig. 2:**
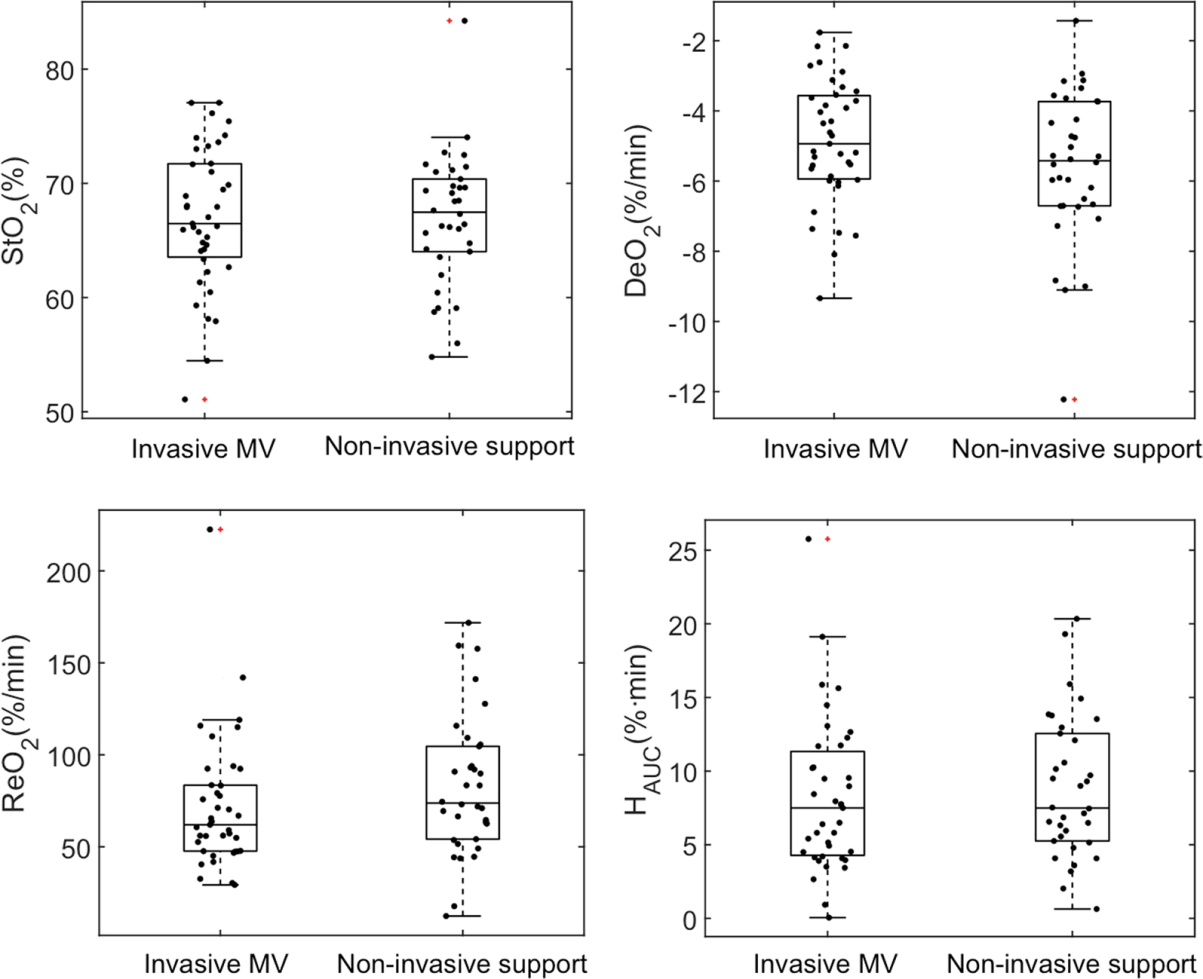
Box-plot and individual data points showing microcirculatory alterations in IRCU/ICU patients according to respiratory support. The distribution of StO_2_ parameters did not differ when categorizing the patients according to receiving invasive mechanical ventilation (53%) or non-invasive respiratory support (47%). The most frequent non-invasive support was high-flow nasal cannula (32%), followed by Venturi mask (11%), and non-invasive mechanical ventilation (4%)

Among the COVID-19 patients studied in the ICU, 39 (53%) were receiving invasive MV at inclusion, all of whom also received sedative agents, and 12 (29%) required norepinephrine infusion to maintain mean arterial pressure (MAP). Patients with non-invasive respiratory support (34) were mainly (68%) receiving high flow nasal cannula therapy (HFNC). No significant differences were observed in hemodynamic variables, or oxygenation when comparing the different respiratory support groups, except for the use of sedation exclusively in the MV group (with or without vasopressors). When analyzing the microcirculatory status according to the degree of respiratory support, no significant differences were observed among groups (Table 2; Fig. 2).

**Table 2:** Main characteristics of severe COVID-19 patients classified according to respiratory support at inclusion

	Invasive mechanical ventilation (*n* = 39)	Non-invasive respiratory support (*n* = 34)
Age (years)	59 ± 12	59 ± 14
Gender (male), *n* (%)	28 (72)	23 (68)
BMI	30 ± 5	30 ± 6
Days from hospitalization (*n*)	5 ± 4	5 ± 4
Days from IRCU/ICU admission (*n*)	4 ± 3	3 ± 3
*Pre-existing comorbidities* (*n*, %)		
Hypertension	18 (46)	15 (44)
Diabetes mellitus	8 (21)	9 (26)
Smoker	7 (18)	11 (32)
COPD	2 (5)	5 (15)
*Respiratory support* (*n*, %)		
Mechanical ventilation	39 (100%)	–
HFNC	–	23 (68%)
NIV	–	3 (9%)
Venturi mask	–	8 (23%)
Sedative agents (*n*, %)	39 (100)	0
NMBA (*n*, %)	17 (44)	0
Vasopressor support (*n*, %)	12 (29)	0
Temperature (°C)	36.2 ± 0.9	36.3 ± 0.5
HR (beats/min)	74 ± 15	82 ± 16*
MAP (mmHg)	83 ± 10	89 ± 13*
RR (resp/min)	22 ± 4	21 ± 6
FiO_2_ (%)	53 ± 21	56 ± 20
PaO_2_ (mmHg)	82 ± 14	–
PaCO_2_ (mmHg)	45 ± 8	–
pH	7.38 ± 0.07	–
SpO_2_ (%)	96 ± 2	93 ± 5*
PF ratio	174 ± 54	–
SF ratio	204 ± 64	200 ± 98
Hb (g/dL)	12.4 ± 2.0	13.7 ± 1.8*
D-Dimer (ng/mL)	5528 ± 8833	7499 ± 20,109
Ferritin (ng/mL)	1786 ± 918	1201 ± 993*
StO_2_ (%)	67 ± 6	67 ± 6
THC (μM/L)	41 ± 13	44 ± 18
DeO_2_ (%/min)	− 4.9 ± 1.8	− 5.6 ± 2.1
ReO_2_ (%/min)	72 ± 36	83 ± 38
Hyperemia AUC (U)	8.2 ± 5.2	8.8 ± 4.8

BMI, body mass index; IRCU, Intermediate Respiratory Care Unit; ICU, Intensive Care Unit; COPD, chronic obstructive pulmonary disease; HFNC, high-flow nasal cannula; NIV, non-invasive ventilation; NMBA, neuromuscular blocking agents; HR, heart rate; MAP, mean arterial pressure; RR, respiratory rate; FiO_2_, fraction of inspired oxygen; PaO_2_, arterial partial pressure of oxygen; PaCO_2_, arterial partial pressure of carbon dioxide; SpO_2_, pulse-oximetric oxygen saturation; PF ratio, PaO_2_ to FiO_2_ ratio; SF ratio, SpO_2_ to FiO_2_ ratio; Hb, hemoglobin; StO_2_, tissue oxygen saturation; THC, tissue hemoglobin concentration; DeO_2_, deoxygenation rate; ReO_2_, reoxygenation rate; AUC, area under the curve

^*^p < 0.05

**Fig. 3:**
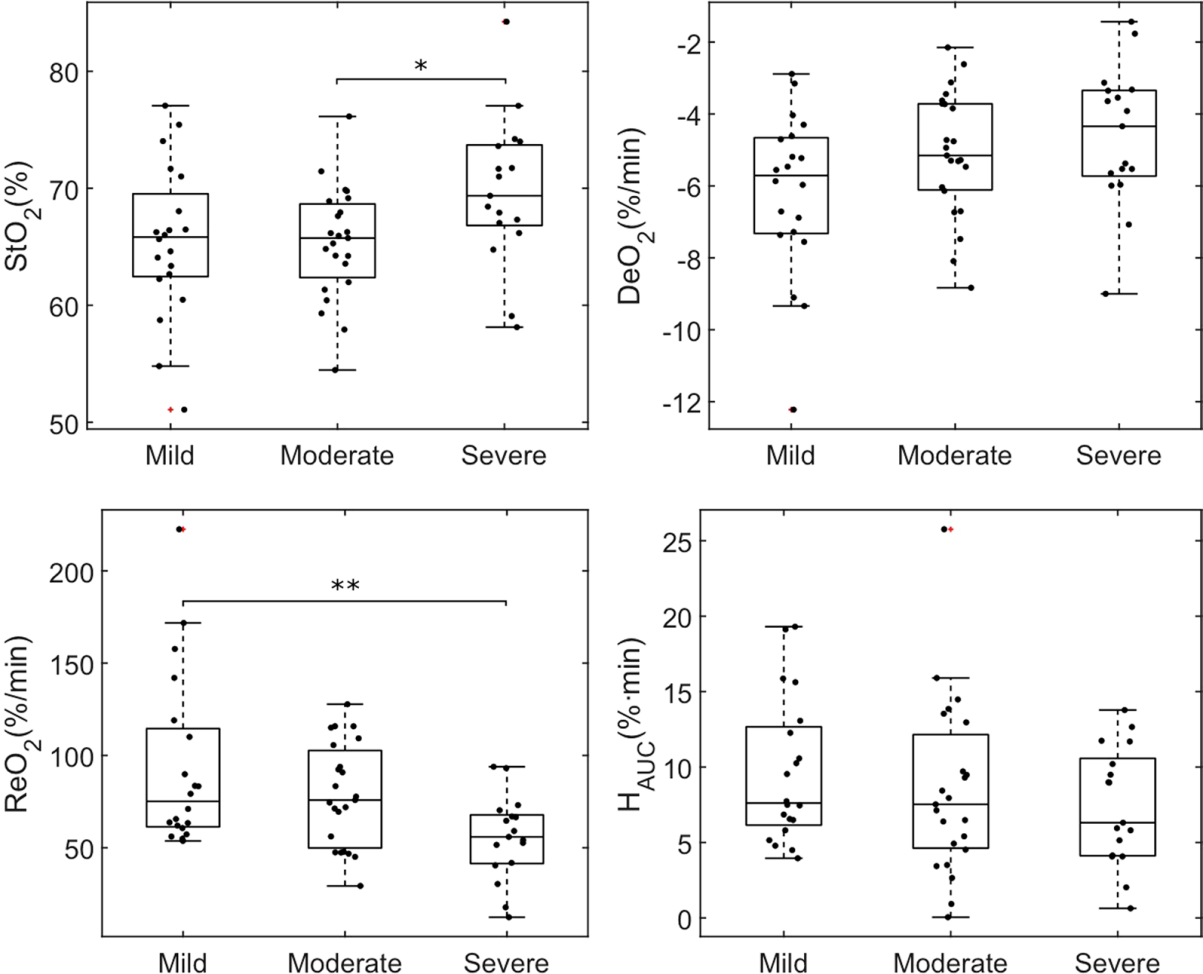
Box-plot and individual data points showing microcirculatory alterations in IRCU/ICU patients according to the severity of ARDS. The distribution of StO_2_ and ReO_2_ was significantly different among patients according to the severity of ARDS. A post hoc Tukey comparison showed that severe ARDS patients had higher StO_2_ values as compared to moderate ARDS, and lower ReO_2_ values, as compared to mild ARDS. The distribution of DeO_2_ among groups did not reach statistical significance (*p* = 0.053)

When analyzing the severity of ARDS, patients were classified as mild (33%), moderate (39%), and severe (28%). We observed significant differences in the distribution of StO_2_ (65.5 ± 6.5% vs 65.3 ± 4.8% vs 69.7 ± 6.3% for mild, moderate and severe, *p* = 0.04) and ReO_2_ (93 ± 47%/min vs 79 ± 28%/min vs 56 ± 23%/min, for mild, moderate and severe, *p* < 0.01) values among groups, with higher StO_2_ values and lower ReO_2_ values in severe ARDS patients (Fig. 3).

Binary correlation tests showed that SF ratio significantly correlated with ReO_2_ (*r* = 0.5, *p* < 0.001), and negatively correlated with baseline StO_2_ (*r* =  − 0.3, *p* = 0.02), and DeO_2_ (*r* =  − 0.4, *p* = 0.001). In those patients where simultaneous arterial blood gas tests were available (*n* = 38), PF ratio significantly correlated with ReO_2_ (*r* = 0.4, *p* = 0.01), and with the SF ratio (*r* = 0.9, *p* < 0.001). SF ratio also inversely correlated with age (*r* =  − 0.3, *p* = 0.02) while no correlations with BMI or measured inflammatory markers were observed.

Simple linear regression showed that SF ratio was associated with age, chronic hypertension, StO_2_, DeO_2_, and ReO_2_. These variables were used to construct multiple linear regression models, also including BMI as an independent variable, despite not reaching statistical significance in the univariate analysis (*p* = 0.09). Only the microcirculatory status remained independently associated with SF ratio (Table 3).

**Table 3:** Multivariate linear regression for association with SF ratio

	*B*	*β*	(95% CI)	*p*
Age	− 1	− 0.16	(− 2.6, 0.6)	0.2
Hypertension	− 36	− 0.23	(− 75, 3)	0.07
BMI	− 1.7	− 1.1	(− 5.4, 1.9)	0.3
StO_2_	− 3	− 0.23	(− 6, − 0.6)	0.05
ReO_2_	0.7	0.33	(0.2, 1.2)	< 0.01

BMI, body mass index; StO_2_, tissue oxygen saturation; ReO_2_, reoxygenation rate

## Discussion

The main result of our study is that severe COVID-19 patients admitted in the ICU showed altered microcirculatory status in the peripheral muscle, and the degree of such alterations correlated with the severity of the respiratory disease. To our knowledge, this is the first study evaluating the peripheral microcirculation in critically ill COVID-19 patients at the bedside using non-invasive NIRS.

Our findings reinforce the idea of a systemic microvascular involvement in severe COVID-19 patients, and further supports the association between the degree of endothelial dysfunction (ED), expressed as poor microvascular reactivity, and the severity of the disease, including the respiratory involvement [9]. There is growing evidence supporting the role of ED in the time course of severe COVID-19. Several mechanisms have been proposed to contribute to ED, including direct endothelial cell damage produced by the virus, down-regulation of the angiotensin-converting enzyme 2 (ACE2) receptors, the inflammatory response of the host, or even as a results of tissue hypoxia in the setting of severe hypoxemia, among others [17–23]. Some authors suggest that COVID-19 might be considered an endothelial disease [8, 19, 20], and those patients with more severe forms, probably due to individual predisposition, will develop not only respiratory disease, but also systemic disease, with generalized ED, coagulopathy, disseminated intravascular coagulation (DIC), and multi-organ failure [22, 23]. Our study does not provide insights in the mechanisms of ED, but confirms that microvascular reactivity, as a surrogate of endothelial function, can be properly evaluated and monitored in severe COVID-19 patients by means of non-invasive near-infrared spectroscopy technologies. Of note, the technology does not allow for the evaluation of other aspects of the endothelium that might be altered in COVID-19, such as the barrier function or the control of the coagulation.

ED also plays a key role in ARDS induced by other causes, such as sepsis or other respiratory viral infections. In fact, microvascular reactivity alterations, evaluated by means of NIRS, have been associated with poor prognosis in a mixed population of ARDS patients [24], and in a small series of patients with acute lung injury due to influenza AH1N1 [25]. Such observations, and ours, would be complementary, pointing towards the value of evaluating microvascular reactivity by means of NIRS technologies, independently of the underlying disease that led to endothelial damage. Accordingly, microvascular reactivity evaluation, as a reflection of endothelial function, might be a useful tool for prognostic purposes in several critical conditions. Whether microvascular reactivity is associated with poor prognosis, in terms of mortality, in COVID-19 patients is currently being investigated in a large multicenter trial (NCT04689477; Hemocovid19-project.org). This study is an interim report from the larger trial. Since ED is an old companion of several critical conditions, the larger trial will also address the issue of whether there are differences in the degree of alterations in microvascular reactivity in COVID and non-COVID populations, and its impact on outcomes. Clearly, our findings on microvascular reactivity alterations are not expected to be limited to COVID-19 patients, nor useful as a specific diagnostic tool for detecting COVID-19, but only as a quantification of the involvement of the systemic endothelium in the process of the disease. Furthermore, the value of microvascular reactivity alterations in heterogeneous populations may notably differ from our findings.

To date, large series of COVID-19 patients have demonstrated that some comorbidities such as hypertension, diabetes, cardiovascular diseases, and obesity are associated with higher risk of developing severe disease [26–28]. Such observations have been linked to increased ACE2 expression in those conditions, and therefore an increased vulnerability of the endothelial cells to the action of the virus. Although our sample size might be limited, our regression analysis points towards the importance of endothelial function evaluation, independent of any underlying comorbidities.

In addition to compromised microvascular reactivity, we also observed significant impairments in local tissue metabolic rate, as reflected by impaired DeO_2_ values in COVID-19 patients, as compared to healthy volunteers. Such alterations, also observed in other conditions such as sepsis [10–12], reflect the inability of the explored area to properly use the oxygen available, and might be caused by microvascular thrombosis, tissue edema, and/or mitochondrial dysfunction. We cannot distinguish the underlying mechanism, but note that it does occur, and that it reveals microvascular disease.

An interesting finding of our study was that no differences in microcirculatory involvement were detected when comparing patients receiving invasive MV and those receiving non-invasive respiratory support. Such findings may be surprising, since patients receiving MV were likely more severe. In this study, we do have detected an association between the degree of ARDS severity according to the degree of hypoxemia, but we have not explored the association with other respiratory parameters, such as respiratory mechanics. The decision to intubate a patient was not protocolized/standardized in our study, and the attending physicians in each participating center made independent clinical decisions. Therefore, a certain degree of variability in the management of hypoxemia among centers is expected. The approach to managing hypoxemia in COVID-19 patients is a complex debate. Some authors propose a less invasive approach, since many patients exhibit what has been named "happy" or "silent hypoxemia" [29–31]. The decision to intubate COVID-19 patients based only on a certain degree of hypoxemia has been questioned, but the truth is that it is still a current practice in the clinical scenario, and even in many randomized controlled trials. For instance, in a recent multicenter trial analyzing the use of awake prone positioning, mortality did not differ between patients treated with high-flow nasal cannula and patients that required mechanical ventilation [32], highlighting the issue that the use of mechanical ventilation might not be only associated with the severity of the illness, but also to different practices among clinicians when facing the management of hypoxemia in COVID-19 patients. On that behalf, we hope our larger trial will provide more relevant information on this subject.

Furthermore, the lack of differences in microvascular reactivity between invasive and non-invasive MV are important in order to rule out the effect of drugs, such as sedative agents or norepinephrine, as the only explanation to peripheral microcirculatory alterations in ARDS patients. Sedation (deep sedation) might appear as one of the causes of altered microcirculation in COVID, but we already observed equivalent alterations in awake patients receiving HFNC or Venturi mask, without apparent cardiovascular problems. To date, microcirculatory impairment has been extensively associated with hemodynamic alterations, such as in septic shock [8–10]. Of note, none of the studied patients showed increased plasma lactate levels, and only twelve patients were receiving vasopressors for maintaining adequate blood pressure values.

### Study limitations

The primary limitation of this study is the restriction of enrollment to patients with severe forms of COVID-19, and therefore our results might not be valid for mild or moderate forms of the disease, i.e., not requiring intensive care support. In fact, in those patients with less severe presentations of COVID-19, systemic involvement might be limited, and endothelial dysfunction is lower [8], and thus, peripheral microvascular reactivity might appear within normal ranges. Moreover, no measurements prior to IRCU/ICU admission were available, and thus, whether ED precedes respiratory deterioration cannot be deduced. On that point, a small study showed that elevated angiopoietin-2 levels, as a marker of endothelial activation, measured early in the emergency department, were associated with the need for ICU admission [33]. Additional studies should explore the value of early microvascular reactivity evaluation in order to detect those patients with mild forms of the disease who might further deteriorate.

Secondly, arterial blood gas analysis was only computed in MV patients, and the utilization of SpO_2_/FiO_2_ may have some limitations, namely peripheral disturbances, and its lack of sensitivity to take into account the presence of hyperoxemia. However, since many patients included in the trial were receiving non-invasive therapies, and did not have an arterial line, SpO_2_/FiO_2_ was taken as a surrogate of PaO_2_/FiO_2_ in order to reflect respiratory involvement. Although a linear correlation between such parameter and NIRS-derived variables might be affected by the exposed limitations, SpO_2_/FiO_2_ has been previously validated for classifying the severity of ARDS [16]. In addition, in those patients where PaO_2_ was also obtained, the correlation between PaO_2_/FiO_2_ and SpO_2_/FiO_2_ was strong (*r* = 0.9). The lack of peripheral disturbances observed in our population (no relevant cardiovascular issues in our sample) might also account for the strong relationship between these two parameters, but that might not be the case for other clinical scenarios, such as critical conditions with severe hemodynamic impairment.

In our study, most patients were already receiving treatments with potential effects on the microcirculation, such as heparin or corticosteroids. Regrettably, our design does not allow for evaluating the impact of such therapies on the microcirculation.

Finally, this preliminary data was not powered for mortality assessment, but rather to detect microcirculatory alterations in COVID-19 patients, compared to healthy volunteers. The observed association with SF ratio points towards a potential prognostic value when referred to mortality [34, 35]. On that behalf, a larger trial is being conducted in order to test the association between impaired microvascular reactivity and mortality in COVID-19 patients admitted to the IRCU/ICU.

## Conclusion

Severe COVID-19 patients admitted to the IRCU/ICU due to hypoxemic respiratory failure showed alterations in the systemic microcirculation. Such alterations, and mainly impairment in microvascular reactivity, were associated with the severity of ARDS and were not explained by the use of sedative agents or vasopressors. Whether these alterations have prognostic implications deserves further evaluation.

## Supplementary Information

**Additional file 1**. Depiction of the StO_2_-parameters obtained as results of the vascular occlusion test (VOT).

## Data Availability

Data will be made available by the corresponding author upon reasonable request.

## Abbreviations

BMI: Body mass index
COPD: Chronic obstructive pulmonary disease
DeO_2_: Deoxygenation rate
ED: Endothelial dysfunction
FiO_2_: Fraction of inspired oxygen
HFNC: High-flow nasal cannula
HR: Heart rate
ICU: Intensive Care Unit
IRCU: Intermediate Respiratory Care Unit
MAP: Mean arterial pressure
NIV: Non-invasive mechanical ventilation
RR: Respiratory rate
SF ratio: SpO_2_ to FiO_2_ ratio
SpO_2_: Pulse-oximetric oxygen saturation
StO_2_: Tissue oxygen saturation
ReO_2_: Reoxygenation rate
THC: Tissue hemoglobin concentration
