# Supplementary material for: Peripheral microcirculatory alterations are associated with the severity of acute respiratory distress syndrome in COVID-19 patients admitted to intermediate respiratory and intensive care units
Source: Crit Care. 2021 Nov 8;25:381. doi: 10.1186/s13054-021-03803-2 (PMC8575160; doi:10.1186/s13054-021-03803-2)
Supplement: Supplementary file 1 — Additional file 1. Depiction of the StO2-parameters obtained as results of the vascular occlusion test (VOT). [file 13054_2021_3803_MOESM1_ESM.docx]

**Additional file 1**

**VOT variables calculation**


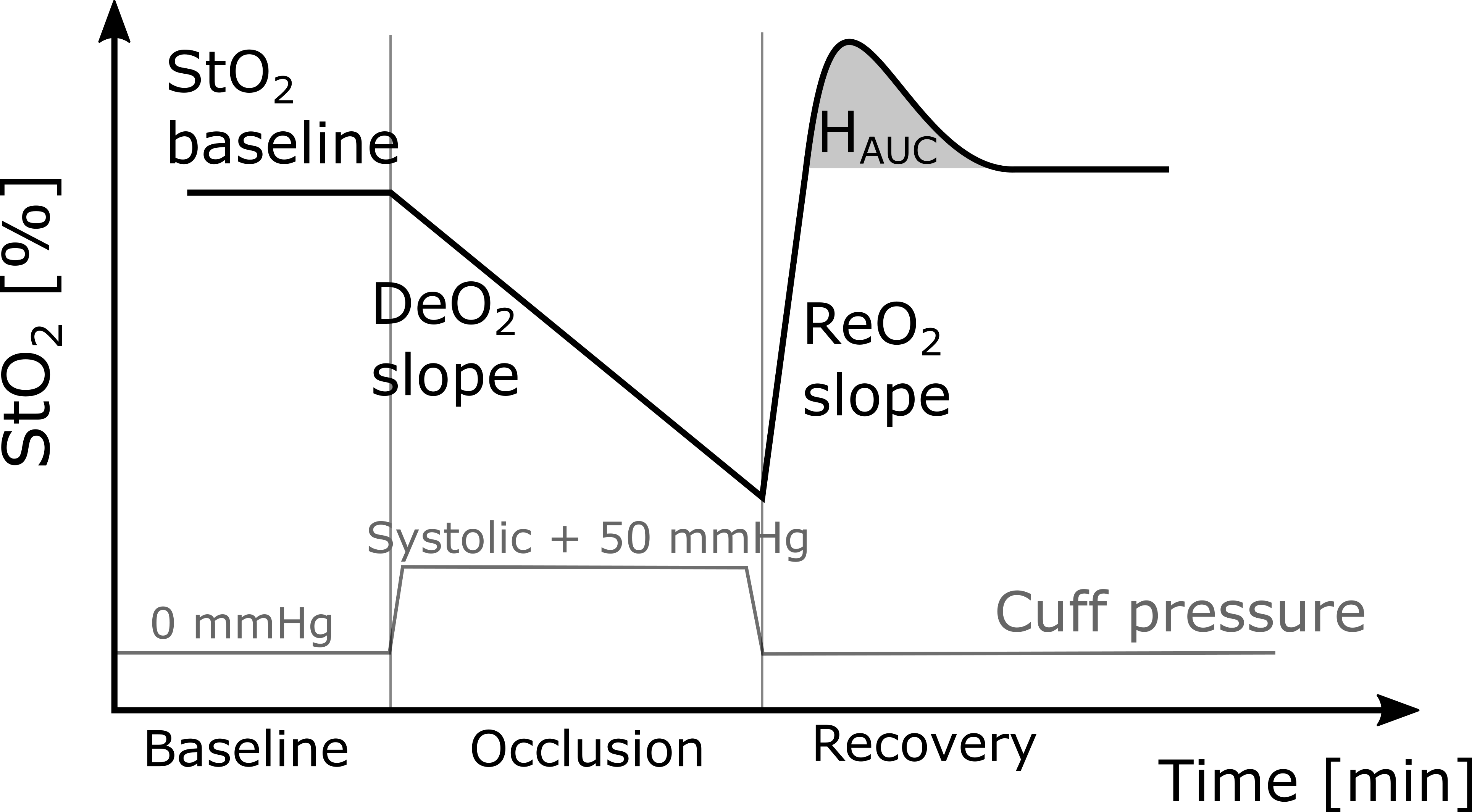


**StO_2_** [%]: Directly measured by the NIRS device

**THC** [µM/L]: Directly measured by the NIRS device

**DeO_2_** [%/min]: The deoxygenation slope has been calculated by linearly fitting the curve StO_2_ [%] vs. Time [min] from the instant the occlusion started up to one minute after the occlusion started

**ReO_2_** [%]: The reoxygenation slope has been calculated by linearly fitting the curve StO_2_ [%] vs. Time [min] from the instant the occlusion ended up to the instant the saturation reached the baseline value.

**H_AUC_** [%∙min]: The hyperemic response has been calculated as the area defined by the hyperemic peak and the recovery baseline StO_2_ level
